# Chemoproteomics enables identification of coatomer subunit zeta‐1 targeted by a small molecule for enterovirus A71 inhibition

**DOI:** 10.1002/mco2.587

**Published:** 2024-06-05

**Authors:** Xiaoyong Li, Jin Zhang, Yaxin Xiao, Hao Song, Yuexiang Li, Wei Li, Ruiyuan Cao, Song Li, Yong Qin, Chu Wang, Wu Zhong

**Affiliations:** ^1^ Key Laboratory of Drug‐Targeting and Drug Delivery System of the Education Ministry, Sichuan Engineering Laboratory for Plant‐Sourced Drug, and Sichuan Research Center for Drug Precision Industrial Technology, West China School of Pharmacy Sichuan University Chengdu China; ^2^ National Engineering Research Center for the Emergence Drugs Beijing Institute of Pharmacology and Toxicology Beijing China; ^3^ College of Chemistry and Molecular Engineering Peking University Beijing China

**Keywords:** chemoproteomics, coatomer subunit zeta‐1, enterovirus A71, marinopyrrole A derivate

## Abstract

Human enterovirus A71 (EV‐A71) is a significant etiological agent responsible for epidemics of hand, foot, and mouth disease (HFMD) in Asia‐Pacific regions. There are presently no licensed antivirals against EV‐A71, and the druggable target for EV‐A71 remains very limited. The phenotypic hit 10,10′‐bis(trifluoromethyl) marinopyrrole A derivative, herein termed MPA‐CF_3_, is a novel potent small‐molecule inhibitor against EV‐A71, but its pharmacological target(s) and antiviral mechanisms are not defined. Here, quantitative chemoproteomics deciphered the antiviral target of MAP‐CF_3_ as host factor coatomer subunit zeta‐1 (COPZ1). Mechanistically, MPA‐CF_3_ disrupts the interaction of COPZ1 with the EV‐A71 nonstructural protein 2C by destabilizing COPZ1 upon binding. The destruction of this interaction blocks the coatomer‐mediated transport of 2C to endoplasmic reticulum, and ultimately inhibits EV‐A71 replication. Taken together, our study disclosed that MPA‐CF_3_ can be a structurally novel host‐targeting anti‐EV‐A71 agent, providing a structural basis for developing the COPZ1‐targeting broad‐spectrum antivirals against enteroviruses. The mechanistic elucidation of MPA‐CF_3_ against EV‐A71 may offer an alternative COPZ1‐involved therapeutic pathway for enterovirus infection.

## Graphical abstract

MPA‐CF_3_ destabilizes coatomer subunit zeta‐1 (COPZ1) upon binding and disrupts the interaction of COPZ1 with the EV‐A71 2C protein, resulting in blocking of coat protein complex I (COPI) coat‐mediated transport of 2C to ER and subsequent inhibition of EV‐A71 replication.

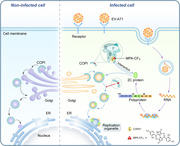

## 1 INTRODUCTION

Human enterovirus A71 (EV‐A71) belongs to enterovirus species A that are among the *Enterovirus* genus in the *Picornaviridae* family. As a nonenveloped, positive‐sense, single‐stranded RNA virus,^1^ EV‐A71 is one of the most important pathogens that cause hand, foot, and mouth disease (HFMD) in Asia‐Pacific regions and some European areas and mainly infects children under the age of 5 years.^2^, ^3^, ^4^ The majority of clinical syndromes of EV‐A71 infection are mild and self‐limited. However, EV‐A71 is a highly neurotropic virus and EV‐A71 infection can develop various neurological complications, including brainstem encephalitis, aseptic meningitis, acute flaccid paralysis, and neurogenic pulmonary edema, in some severe cases.^5^, ^6^ Although three inactivated EV‐A71 vaccines have been licensed in China, clinical cross protection against other genogroups has not been fully demonstrated, and more appropriate animal data should be evaluated to support the breadth of protection.^7^, ^8^, ^9^ Therefore, small molecule drugs with novel structure are particularly attractive for complex variants of EV‐A71.

Compared with target‐based drug discovery, phenotypic screening for small molecule drugs has resurged, underlying the contribution of phenotypic approaches to discover lead compounds.^10^ In combination with modern target deconvolution and elucidation of mechanisms of action (MOAs), phenotypic approaches greatly advance effective drug discovery. We previously reported 10,10′‐bis(trifluoromethyl) marinopyrrole A derivative (termed MPA‐CF_3_) with excellent antiviral activity against EV‐A71 and coxsackievirus A16 through phenotypic screening.^11^ In this study, we constructed and synthesized a clickable photoaffinity probe of MPA‐CF_3_ to globally explore the antiviral targets in EV‐A71‐infected human rhabdomyosarcoma (RD) cells. Chemoproteomics disclosed that the primary target of MPA‐CF_3_ is the host factor coatomer subunit zeta‐1 (COPZ1), which was further validated by biophysical and biochemical assays. We finally elucidated the MOA of MPA‐CF_3_ against EV‐A71. Our findings demonstrate MPA‐CF_3_ as a host‐targeting antiviral agent and provide mechanistic insights to explain the anti‐EV‐A71 activity, which may enable MPA‐CF_3_ to be a starting lead for COPZ1‐targeting rational drug design in future.

## 2 RESULTS

### 2.1 Design and synthesis of the photoaffinity probe MPA‐P

**FIGURE 1:**
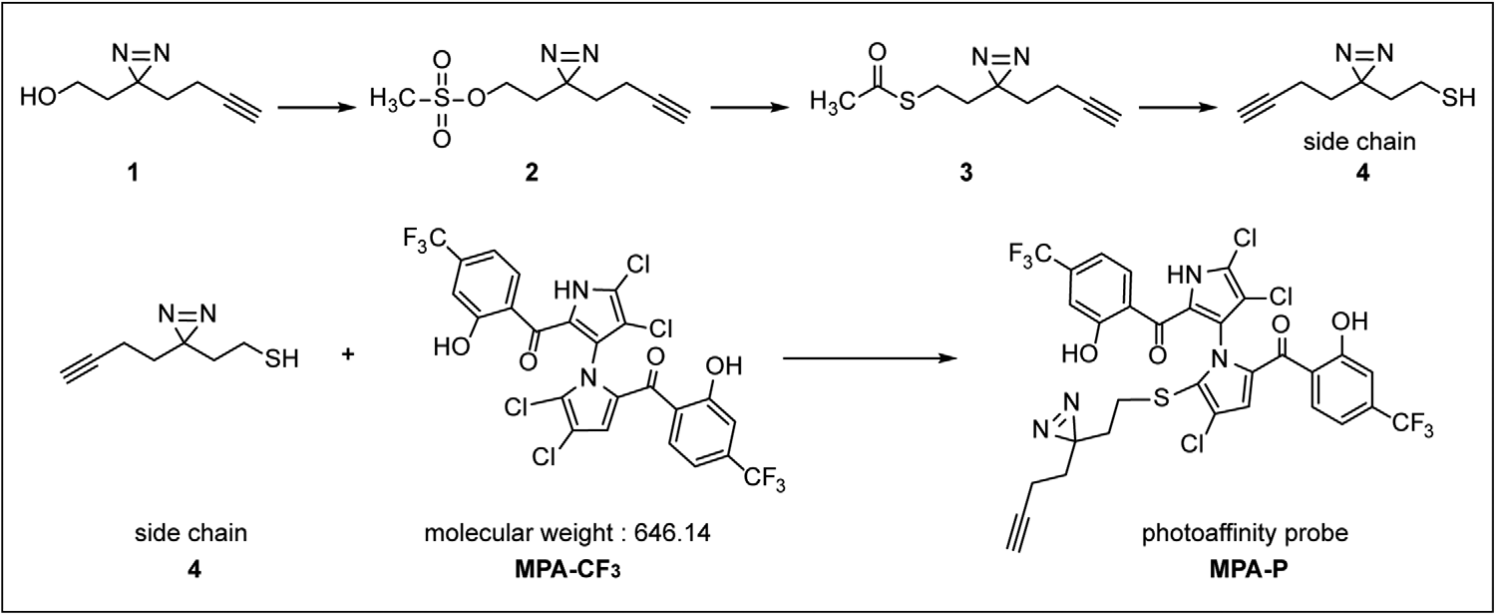
Synthetic routes of photoaffinity probe MPA‐P. Starting from the minimalist photo‐crosslinker **1**, a two‐step substitution reaction and a reduction reaction were performed to generate the side chain **4**, which was introduced into the 5′‐position of the pyrrole moiety of 10,10′‐bis(trifluoromethyl) marinopyrrole A derivative (MPA‐CF_3_) to afford MPA‐P.

To identify the potential anti‐EV‐A71 targets of MPA‐CF_3_, it is crucial to develop a pharmacologically active chemical probe based on MPA‐CF_3_. We previously synthesized a series of 5′‐sulfhydryl derivatives of MPA‐CF_3_ and conducted extensive structure–activity relationship studies for their anti‐EV‐A71 activities. The result suggested that the antiviral activities were highly associated with the sulfhydryl aliphatic chains (R‐SH) or mercapto heterocycles at the 5′‐position of the pyrrole moiety.^11^ We followed this path that led us to construct a sulfhydryl side chain bearing a diazirine group and a clickable alkyne handle, which was then installed to the 5′‐position of pyrrole moiety of MPA‐CF_3_ to generate a photoaffinity probe MPA‐P (Figure 1 and Method S1, Supporting Information).

### 2.2 MPA‐P retains potent activity against EV‐A71 and can effectively label the proteome in EV‐A71‐infected RD cells

**FIGURE 2:**
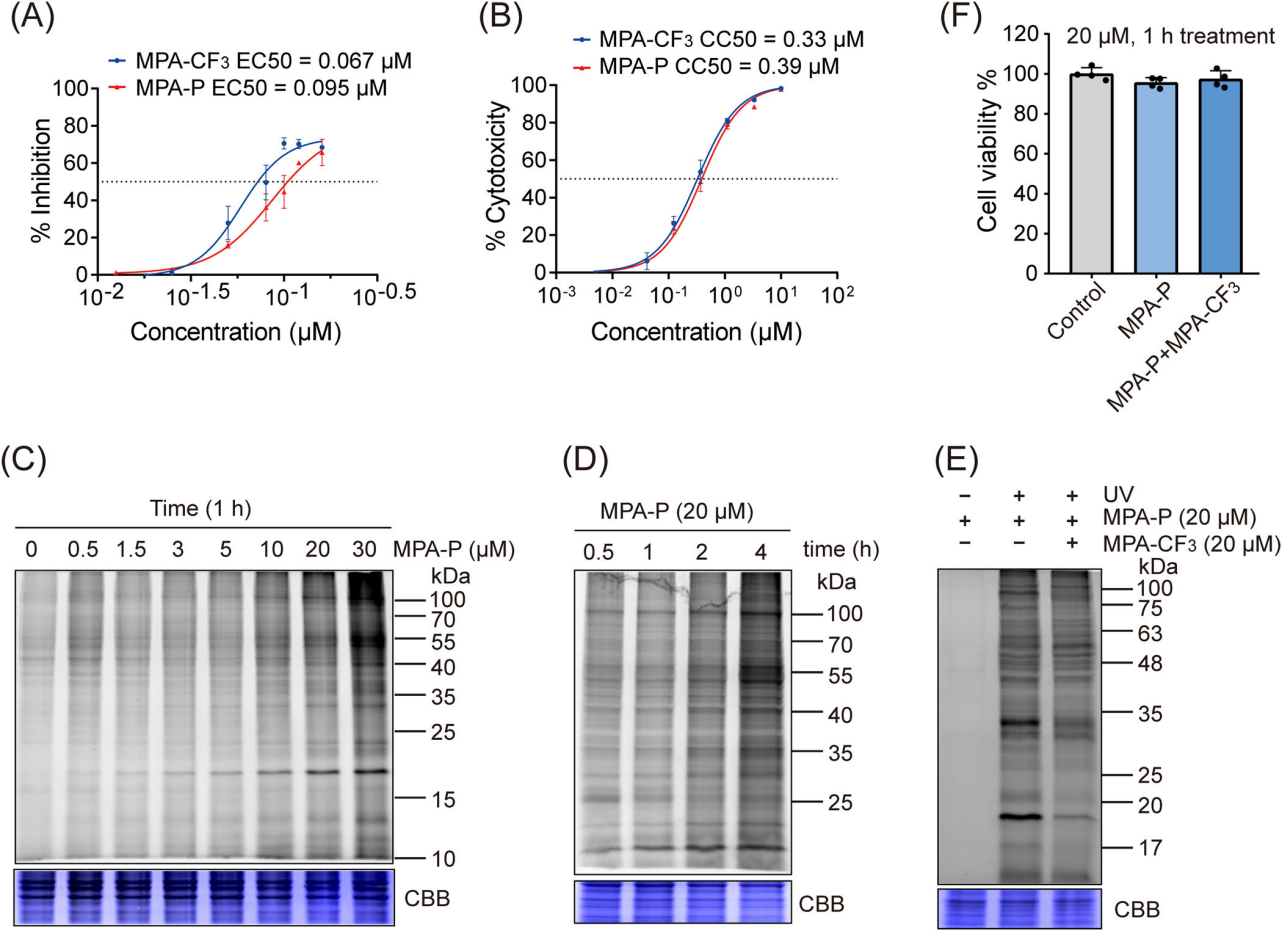
Antiviral activity determination and labeling performance evaluation of MPA‐P. (A) EC50 determination of MPA‐CF_3_ and MPA‐P in EV‐A71‐infected RD cells. (B) CC50 analysis of MPA‐CF_3_ and MPA‐P in RD cells. (C and D) Concentration‐ and time‐dependent labeling of MPA‐P in EV‐A71‐infected RD cells by in‐gel fluorescence visualization. (E) Competition labeling of MPA‐P with the competitor MPA‐CF_3_ in EV‐A71‐infected RD cells. (F) Cell viability assay of MPA‐P treatment or cotreatment with MPA‐CF_3_ for 1 h in EV‐A71‐infected RD cells. Data (A), (B), and (F) were obtained from four biological replicates. CBB, Coomassie brilliant blue staining.

To perform quantitative chemoproteomics investigation, we first determined 50% effective concentration (EC50) and 50% cytotoxic concentration (CC50) of MPA‐P in RD cells. The result showed that the EC50 and CC50 are closely comparable to those of the parent compound MPA‐CF_3_ (Figures 2A and B), supporting subsequent labeling in situ. We then evaluated the labeling performance of MPA‐P by in‐gel fluorescence assay in the EV‐A71‐infected RD cells. As shown in Figures 2C and D, a clearly labeled band at 15−25 kDa was observed in a concentration‐ and time‐dependent manner. We next selected 20 µM MPA‐P for competition labeling with the competitor MPA‐CF_3_ (20 µM) with no cytotoxicity within the time frame of 1 h labeling (Figure 2F). The result indicated that MPA‐CF_3_ substantially outcompeted MPA‐P labeling in an in‐gel fluorescence assay (Figure 2E), suggesting that MPA‐P potentially binds to true drug targets in situ as MPA‐CF_3_ does.

### 2.3 Quantitative chemoproteomics identified the target of MPA‐CF_3_ as COPZ1

**FIGURE 3:**
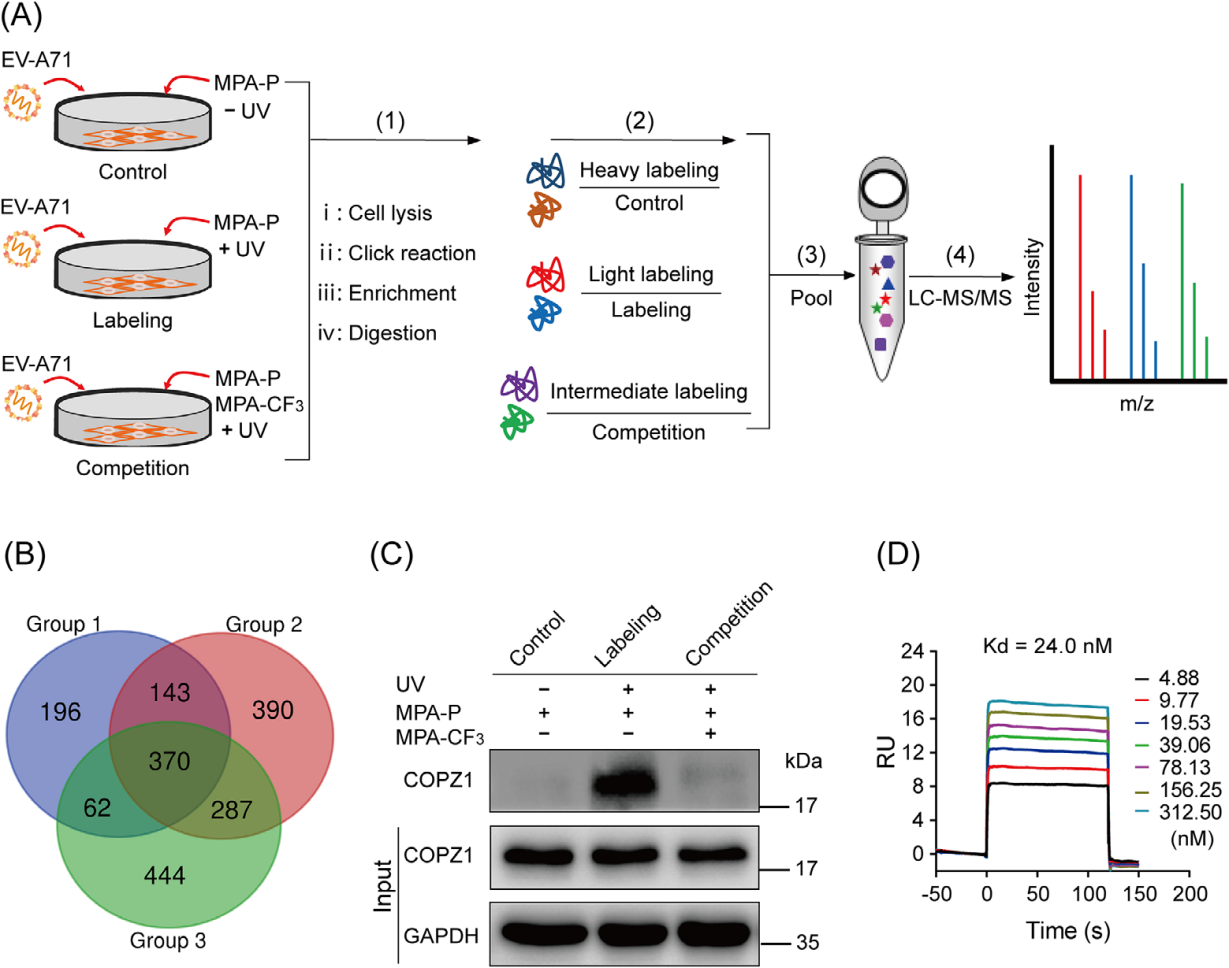
Target identification and validation of MPA‐CF_3_ by quantitative chemoproteomics with stable isotope dimethyl labeling. (A) Overall schematic of the photoaffinity labeling experiments performed to identify MPA‐CF_3_ targets in EV‐A71‐infected RD cells. Three groups of experiments were performed in parallel, including the control group, labeling group and competition group. (B) Venn diagram showing a total of 370 proteins identified from the three groups of isotope dimethyl labeling experiments with set thresholds. (C) Pulldown/western blot analysis of a competition labeling for MPA‐CF_3_ binding protein identification. (D) The binding assay of MPA‐CF_3_ with COPZ1^7‐150^ by SPR analysis.

Inspired by the in‐gel fluorescence results, we subsequently applied a quantitative chemoproteomics approach by stable isotope dimethyl labeling to unravel the global antiviral targets in native infected cells.^12^, ^13^ RD cells were treated with MPA‐P or cotreated with MPA‐CF_3_ after EV‐A71 infection, and subjected to photo‐crosslinking. After cell lysis, the proteins were precipitated, resuspended, subjected to click reaction with biotin–PEG3–azide, and enriched with streptavidin beads. Then, on‐bead tryptic digestion was performed, and the digested peptides were subjected to triplex stable isotope dimethyl labeling, which were defined as heavy labeling (H), medium labeling (M), and light labeling (L) for the control group, competition group, and labeling group, respectively. The three groups of isotopically labeled peptides were mixed and then resolved by liquid chromatography‐tandem mass spectrometry (LC‒MS/MS) (Figure 3A). Finally, a total of 370 proteins were identified in the three replicates after a cutoff of 5.0 was applied for a ratio of L/H labeling and 1.2 for that of L/M labeling (Figure 3B and Dataset S1, Supporting Information). We further searched the proteins within a molecular weight range of 17−20 kDa in the UniProtKB database for that clearly labeled band in the competition labeling experiment and screened out nine proteins (Table S1) for target identification.

Enterovirus infection can induce the rearrangement of cellular membranes to form double‐membrane vesicles known as replication organelles (ROs) on endoplasmic reticulum (ER) to replicate viral RNA.^14^, ^15^ In the early secretory pathway, enteroviruses frequently remodel host membranes and initiate the formation of ROs at ER–Golgi interface.^16^ Coat protein complex I (COPI) vesicles are responsible for cargo trafficking from the Golgi back to the ER.^17^ A previous study reported that COPI is required for EV‐A71 replication and the depletion of its subunit COPZ1 can suppress EV‐A71 infection in RD cells.^18^ These studies suggest that COPZ1 may be a target of MPA‐CF_3_ among the selected nine proteins.

To confirm this speculation, we performed a pulldown/western blot (WB) analysis of competition labeling. According to the steps (i–iii) in Figure 3A, proteins were enriched on the streptavidin magnetic beads and were detected by WB analysis using anti‐COPZ1 antibody. The immunoblotting results confirmed that the labeling intensity of COPZ1 by MPA‐P in EV‐A71‐infected RD cells was significantly weakened in the presence of the competitor MPA‐CF_3_ (Figure 3C), implying that MPA‐CF_3_ specifically binds to COPZ1 in intact cells.

To further measure the binding of MPA‐CF_3_ to COPZ1, we acquired the truncated human COPZ1^17‐150^ from an *Escherichia coli* system,^19^ and conducted an surface plasmon resonance (SPR) experiment to detect the binding interaction between MPA‐CF_3_ and COPZ1. A *K*_d_ value of 24 nM displayed a high binding affinity of MPA‐CF_3_ with COPZ1, demonstrating a strong interaction between MPA‐CF_3_ and COPZ1 (Figure 3D).

### 2.4 Biological engagement of COPZ1 in the inhibition of EV‐A71 replication

**FIGURE 4:**
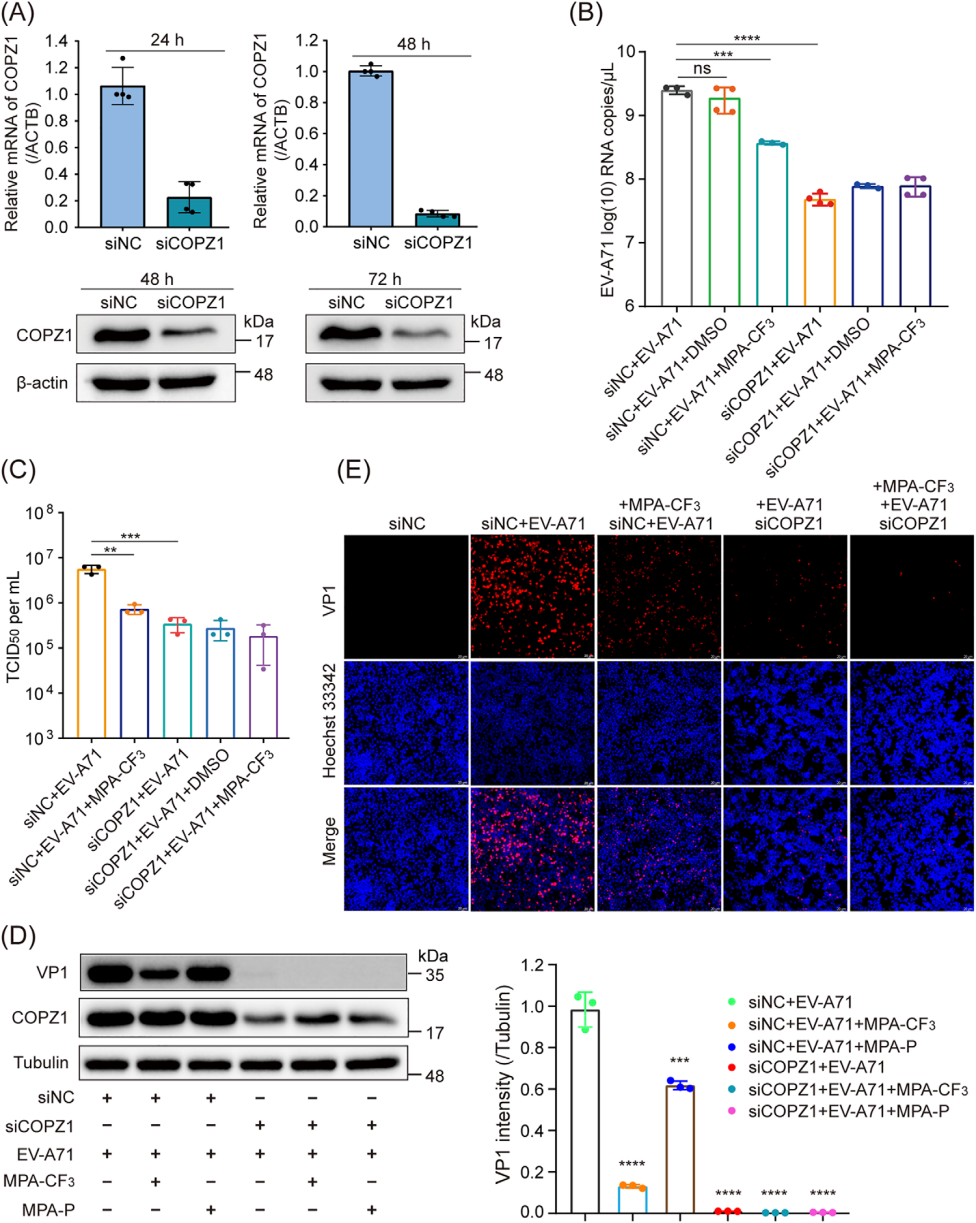
COPZ1 participates in suppression of EV‐A71 replication. (A) Assessment of COPZ1 knockdown using siRNA in RD cells. (Top) Relative mRNA level of COPZ1 quantified by qRT‐PCR at 24 and 48 h posttransfection. (Bottom) Western blot analysis of COPZ1 protein expression at 48 and 72 h posttransfection. (B) Determination of the EV‐A71 RNA copies by qRT‐PCR treated with MPA‐CF_3_ at 72 h posttransfection. (C) TCID_50_ titration of the EV‐A71 progeny yield treated with MPA‐CF_3_ at 72 h posttransfection. (D) (Left) Western blot analysis of the EV‐A71 VP1 and COPZ1 protein expression and (right) the relative intensity analysis of VP1 and COPZ1 treated with MPA‐CF_3_ or MPA‐P at 72 h posttransfection. (E) Immunofluorescence imaging of VP1 protein expression. Scale bars: 20 µm. Data are presented as mean ± SD. Statistical analyses were performed by one‐way ANOVA. **p < 0.01, ***p < 0.001, ****p < 0.0001. ns, no significance. siNC, negative control siRNA; siCOPZ1, siRNA targeting COPZ1.

The phenotypic effect of COPZ1 on EV‐A71 replication was investigated by using small interfering RNAs (siRNAs) in RD cells. After COPZ1 was knocked down by siRNA (Figure 4A), the EV‐A71 RNA copies were significantly reduced compared with that of negative control siRNA treatment, as determined by quantitative real‐time PCR (qRT‐PCR), which was even lower than that of MPA‐CF_3_ treatment (Figure 4B). We next investigated the effect of COPZ1 knockdown or MPA‐CF_3_ treatment on infectious virus generation. 50% tissue culture infectious dose (TCID_50_) titrated from the culture supernatant showed that the EV‐A71 progeny yield was markedly reduced after COPZ1 knockdown, which was similar to that observed with MPA‐CF_3_ treatment (Figure 4C). We further detected the EV‐A71 viral protein production by WB analysis and immunofluorescence imaging. Similar to the reduced viral RNA, COPZ1 knockdown substantially decreased the level of the EV‐A71 capsid protein VP1 in EV‐A71‐infected RD cells. Strikingly, COPZ1 knockdown combined with MPA‐CF_3_ treatment almost completely suppressed VP1 expression. In addition, the probe MPA‐P treatment dramatically decreased the level of VP1, demonstrating that the photoaffinity probe successfully labeled the target (Figure 4D). Immunofluorescence imaging showed that COPZ1 knockdown significantly blocked VP1 expression and can further decrease the fluorescence signal of VP1 when combined with MPA‐CF_3_ treatment (Figure 4E). These results demonstrate that COPZ1 is required for EV‐A71 replication in RD cells and MPA‐CF_3_ binding to COPZ1 can suppress EV‐A71 replication.

### 2.5 Mechanisms of action of MPA‐CF_3_ for the inhibition of EV‐A71 replication

**FIGURE 5:**
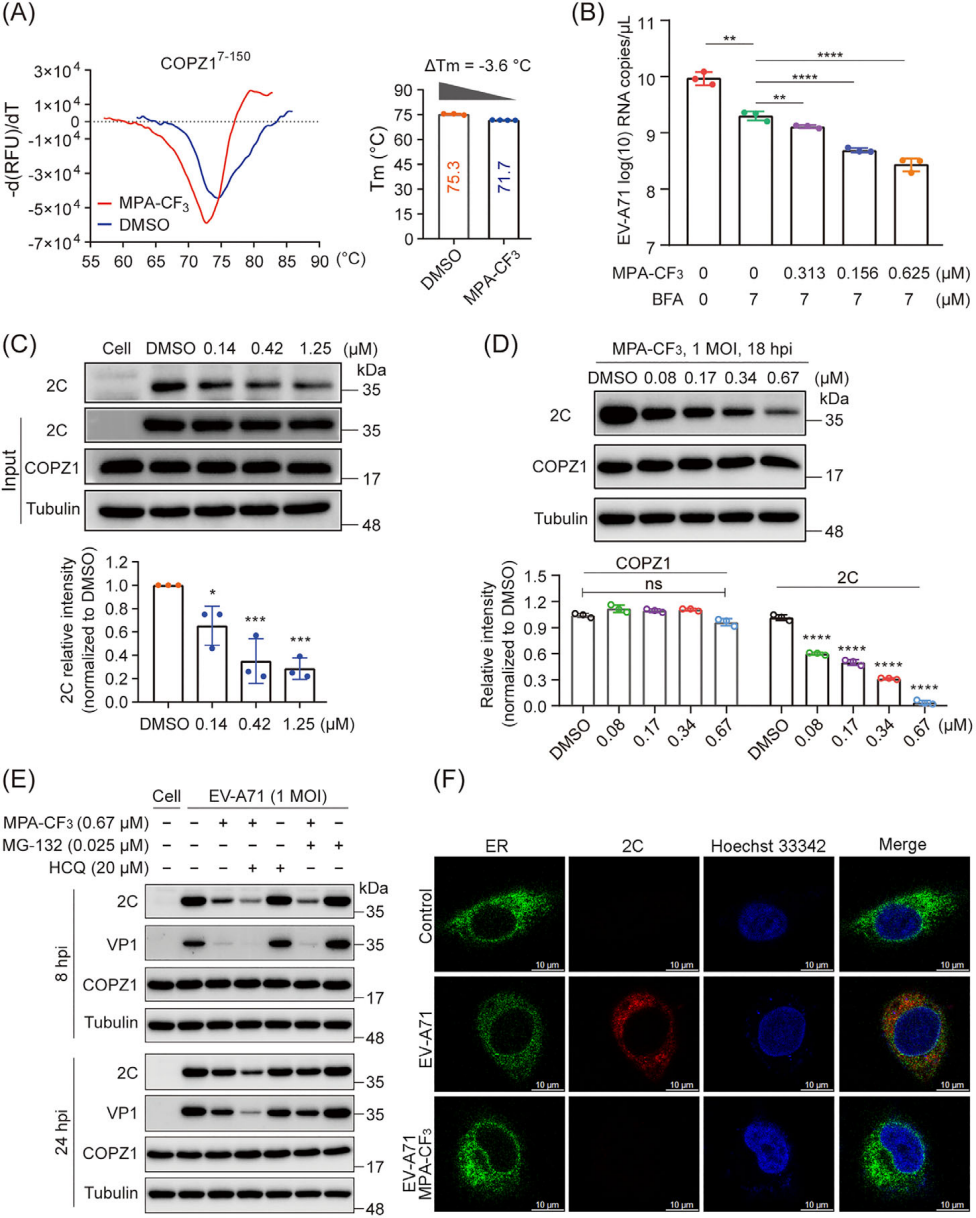
Antiviral mechanisms of MPA‐CF_3_ targeting COPZ1 against EV‐A71. (A) TSA assays of MPA‐CF_3_ binding to the recombinant COPZ1^7‐150^ protein. (Left) Graphs of inflection point of relative fluorescence units against temperature. (Right) Melting temperature (*T*_m_) for COPZ1. Δ*T*_m_ are calculated by subtracting the *T*_m_ of COPZ1 with DMSO treatment from that of MPA‐CF_3_ treatment. (B) Determination of the EV‐A71 RNA copies by qRT‐PCR after 18 hpi. RD cells were treated with brefeldin A (BFA) for 4 h prior to EV‐A71 infection (1.5 h, 1 MOI), followed by MPA‐CF_3_ treatment for 2 h at the indicated concentrations. (C) Co‐immunoprecipitation analysis of the interaction of COPZ1 with the 2C protein in EV‐A71‐infected lysate. (Top) Western blot analysis of the 2C protein precipitated by COPZ1 coincubated with MPA‐CF_3_. (Bottom) The relative intensity analysis of the 2C protein. (D) (Top) Western blot analysis of viral 2C and COPZ1 protein level after 18 hpi. RD cells were pretreated with MPA‐CF_3_ for 2 h and then infected with EV‐A71 for 1.5 h. (Bottom) The relative intensity analysis of COPZ1 and 2C protein. (E) Effects of the lysosome inhibitor hydroxychloroquine (HCQ) and proteasome inhibitor MG‐132 on the degradation of viral 2C and VP1 proteins at the early (left) and late stages (right) of infection. (F) Confocal analysis of the colocalization of the 2C protein with the ER. The red and green channels represent the 2C protein and ER marker Calnexin, respectively. Nuclei were counterstained with Hoechst 33342 (blue). Scale bars: 10 µm. Statistical analyses were performed by one‐way ANOVA. *p < 0.05, **p < 0.01, ***p < 0.001, and ****p < 0.0001. ns, no significance.

To thoroughly dissect the antiviral mechanisms by which MPA‐CF_3_ inhibits EV‐A71 replication, we first performed a fluorescence‐based protein thermal shift assay (TSA)^20^ to explore the interaction of MPA‐CF_3_ with COPZ1. As represented in Figure 5A, the presence of MPA‐CF_3_ had a destabilization effect on COPZ1, as this ligand decreased the melting temperature (*T*_m_) by 3.6°C relative to the reference *T*_m_ in DMSO. This negative thermal shift (Δ*T*_m_ = −3.6°C) implied that MPA‐CF_3_ binding to COPZ1 might impair the functional COPZ1. Typically, the COPI coat (also termed coatomer) resides in the cytosol as a stable heptameric complex. COPZ1, as a subunit of the coatomer, is recruited to Golgi membranes and required for COPI‐coated vesicle assembly.^21^, ^22^ When brefeldin A (BFA)‐treated RD cells were exposed to EV‐A71, COPI recruitment to membranes and the assembly of COPI‐coated vesicles were blocked, subsequently leading to the inhibition of EV‐A71 replication.^18^ We therefore investigated whether MPA‐CF_3_ treatment can interfere with COPI recruitment to membranes upon binding to COPZ1. After treatment with BFA followed by EV‐A71 infection, the subsequent MPA‐CF_3_ treatment under no cytotoxicity condition (Figure S1, left) for 2 h markedly and concentration‐dependently blocked EV‐A71 replication (Figure 5B), indicating that MPA‐CF_3_ binding to COPZ1 prevents the recruitment of COPI‐coated vesicles to membranes and arrests cargo transport essential to EV‐A71 replication.

As COPZ1 can interact with the EV‐A71 nonstructural protein 2C in 293T cell lysates,^18^ we further investigate the effect of MPA‐CF_3_ on the interaction of the two partners by a coimmunoprecipitation (Co‐IP) assay in EV‐A71‐infected cell lysate. Consistent with the reported pulldown of 2C by COPZ1, the presence of MPA‐CF_3_ dose‐dependently dampens the interaction of COPZ1 with 2C (Figure 5C). Because 2C protein plays a pivotal role in regulating the formation of the viral ROs and RNA replication,^23^, ^24^ we then examined the effect of MPA‐CF_3_ on the protein expression of 2C and COPZ1. Pretreatment with MPA‐CF_3_ under no cytotoxicity condition (Figure S1, left) for 2 h prior to EV‐A71 infection dramatically and concentration‐dependently reduced the 2C protein expression, while COPZ1 protein level remained unchanged (Figure 5D). Taken together, these findings demonstrate that MPA‐CF_3_ inhibits EV‐A71 replication by targeting and destabilizing COPZ1 without changing its expression level. As a result, the interaction of COPZ1 with 2C is destroyed, blocking the COPI vesicle‐mediated transport of 2C to ER, where the ROs formed.

**FIGURE 6:**
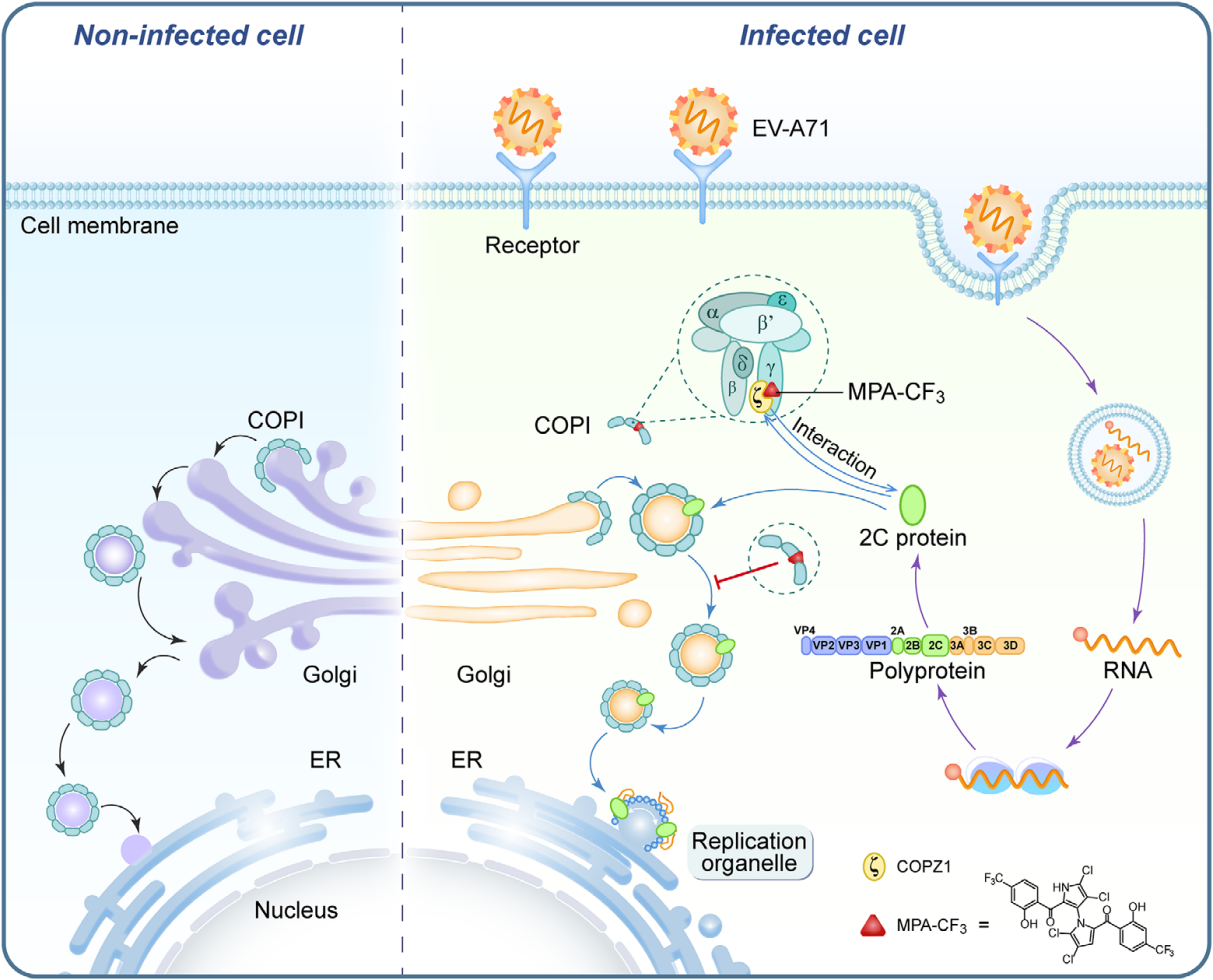
Schematic representation of the proposed mechanism of MPA‐CF_3_ action for the inhibition of EV‐A71 replication. MPA‐CF_3_ specifically binds to COPZ1, a subunit of COPI. Upon binding, MPA‐CF_3_ destabilizes COPZ1 and disrupts the interaction of COPZ1 with the EV‐A71 2C protein in a concentration‐dependent manner. The destruction of this interaction blocks the COPI coat‐mediated transport of 2C to ER and ultimately inhibits EV‐A71 replication.

Since MPA‐CF_3_ disrupts the functional interaction of COPZ1 with 2C in EV‐A71‐infected RD cells, we then determined whether the 2C protein was degraded by the lysosome or ubiquitin‒proteasome system (UPS) in the presence of MPA‐CF_3_. RD cells were pretreated with or without MPA‐CF_3_ for 2 h followed by EV‐A71 infection for 1.5 h, and the lysosome inhibitor hydroxychloroquine (HCQ) or proteasome inhibitor MG‐132 was then added under no cytotoxic conditions (Figure S1, middle and right). EV‐A71 2C and VP1 protein levels were measured at the early (8 hpi) and late stages (24 hpi) of infection. The blots showed that neither HCQ nor MG‐132 could abrogate the 2C and VP1 reduced level in the presence of MPA‐CF_3_ during early or late‐stage infection, implying that lysosomes and the UPS do not participate in the degradation of 2C proteins when exposed to MPA‐CF_3_ (Figure 5E). We further examined the colocalization of the 2C protein with ER at the early stage of infection. Confocal microscopy analysis showed that the transport of the 2C protein to ER was almost blocked in the presence of MPA‐CF_3_, while the 2C protein colocalized with ER in EV‐A71‐infected cells (Figure 5F). These results indicated that the decreased 2C protein mainly resulted from the inhibition of EV‐A71 replication, which occurred after the interaction of 2C protein with COPZ1 was disrupted by MPA‐CF_3_ and COPI coat‐mediated transport of the 2C protein to ER was subsequently blocked. Hence, we proposed the potential antiviral mechanism by which MPA‐CF_3_ inhibits EV‐A71 replication (Figure 6).

## 3 DISCUSSION

EV‐A71‐associated HFMD outbreaks have caused a growing public concern in China, where 15.3 million possible cases of HFMD were documented during 2013−2019.^25^ As a neurotropic pathogen, EV‐A71 infection is often closely linked to severe neurological sequelae and death in children below 5 years old.^26^, ^27^ Understanding the pathogenesis of EV‐A71 and the host restrictive factors in the viral life history is essential for the development of novel therapeutics against EV‐A71 infection. In recent years, several specific direct‐acting antivirals (DAAs) for EV‐A71 have been reported,^28^ including viral capsid protein VP1 inhibitors,^29^, ^30^, ^31^, ^32^ 3C protease inhibitors^33^, ^34^ and 2C helicase inhibitor,^35^ which exhibit promising antiviral activities in vitro or in vivo. These antiviral agents usually lead to the occurrence of drug‐resistant mutants and declining therapeutic efficacies in the case of RNA viruses with high mutation rate.^36^, ^37^ Drugs targeting host proteins show great potential for combating these RNA viruses. By blocking the interaction of the conserved host factors with the viral components involved in the life cycle of enteroviruses, host‐targeting antivirals exhibit broad‐spectrum antiviral activity and a high barrier to resistance.^38^, ^39^ In this context, we screened a structurally novel small‐molecule inhibitor against EV‐A71, MPA‐CF_3_. Chemoproteomics combined with biophysical and biochemical methods identified the specific target of MPA‐CF_3_ as the host factor COPZ1.

COPZ1 is a subunit of coatomer required for the formation of COPI‐coated vesicles that mediate retrograde transport from the cis‐Golgi to the ER or manage intra‐Golgi transport.^22^, ^40^ Although COPZ1 resides in the cytosol in both free and coatomer‐bound forms, unlike the other six coatomer subunits, COPZ1 functions as an integral complex of the coatomer for COPI‐coated vesicle assembly and protein trafficking within the secretory pathway. To date, some investigations of COPZ1 have indicated that COPZ1 is a potential target in tumors,^41^ and a remarkable prognostic marker for multiple human cancers due to its contributions to tumor survival and invasion,^42^, ^43^ but only one report documented that COPZ1 engages in EV‐A71 replication and interacts directly with the EV‐A71 2C protein.^18^ This interaction of host factor with the key viral protein represents a conserved mechanism among different species of enteroviruses, which can be employed to develop broad‐spectrum antivirals for the treatment of enterovirus infections. Our data confirmed that MPA‐CF_3_ disrupts the interaction of COPZ1 with the EV‐A71 2C protein by destabilizing COPZ1 upon binding; as a result, COPI coat‐mediated transport of the 2C protein to ER is prevented and EV‐A71 replication is ultimately suppressed.

The novel small‐molecule based chemical probe is often a powerful tool to explore the interactions between enzymes and substrates (or inhibitors) in the complex biological and pathological systems.^44^ In this study, we used MPA‐CF_3_‐based photoaffinity probe to fish out its key antiviral target in EV‐A71‐infected RD cells, so as to better understand the MOAs of MPA‐CF_3_ against EV‐A71. Although several neuronal cell models were used to study the pathogenesis of EV‐A71 infection in some literatures,^45^, ^46^, ^47^ the well‐established EV‐A71‐infected RD cell model is widely used in drug design, screening, evaluation and MOAs studies.^29^, ^48^, ^49^ The elucidated MOAs based on RD cell in our study completely demonstrated the target engagement of COPZ1 response to MPA‐CF_3_. In addition, the traditional mouse model of EV‐A71 infection uses 2‐ or 3‐day‐old mice, which is hard to observe the significant difference between our drug and the control group, and therefore our current study did not involve in vivo efficacy evaluation of MPA‐CF_3._

In summary, we designed and synthesized a photoaffinity probe from MPA‐CF_3_, a derivative of natural product marinopyrrole A with high potency for EV‐A71. The unbiased quantitative chemoproteomics revealed that the specific target of MPA‐CF_3_ is the host factor COPZ1. Our study demonstrated that MPA‐CF_3_ is a structurally novel host‐targeting anti‐EV‐A71 agent for the first time, which offers an opportunity for the development of effective combination therapies with other DAAs for EV‐A71 cure. Moreover, the elucidated mechanisms of action suggested that MPA‐CF_3_ may serve as a starting lead for COPZ1‐targeting rational design of small molecules against EV‐A71.

## 4 MATERIALS AND METHODS

### 4.1 Cell lines, virus, and compounds

Human RD cells and Vero cells were purchased from the American Type Culture Collection (ATCC; CCL‐136, CCL‐81). All cell lines were cultured in DMEM (Gibco) supplemented with 10% fetal bovine serum (FBS, Gibco) and 1% penicillin/streptomycin (Pen Strep; Gibco) at 37°C under 5% CO_2_. Infected cells were maintained in DMEM supplemented with 2% FBS. The EV‐A71 H strain was provided by the National Engineering Research Center for the Emergency Drug, Beijing Institute of Pharmacology and Toxicology, Beijing. EV‐A71 was propagated in RD cells for cell assays, and viral titers were calculated by plaque assay on RD cells. MPA‐CF_3_ was synthesized in our laboratory. All other reagents were purchased from commercial suppliers.

### 4.2 Synthesis of the photoaffinity probe MPA‐P

As illustrated in Figure 1, compound 1 was prepared according to a previous report.^50^ Compounds 2, 3, and 4 were prepared based on a previous study.^51^ Side chain (compound 4) was reacted with MPA‐CF_3_ to yield the photoaffinity probe MPA‐P.^11^ The detailed preparation of each compound is listed in Method S1, Supporting Information.

### 4.3 Determination of the EC50 and CC50 of MPA‐P

RD cells were seeded in 96‐well plates (Corning; Costar‐3610) at a density of 10,000 cells per well and then incubated for 12 h. For EC50 determination, MPA‐CF_3_ and MPA‐P were added to the wells in a series of indicated concentrations, and DMSO treatment was used as a control. EV‐A71 was inoculated at a multiplicity of infection (MOI) of 0.01 PFU per cell. After 72 h, luminescence was detected using a CellTiter‐Glo Luminescent Cell Viability Assay kit (CTG; Promega) according to the manufacturer's instructions with an EnSpire Multimode Plate Reader (PerkinElmer). For CC50 determination, MPA‐CF_3_ and MPA‐P were added to the wells in a threefold dilution series, and DMSO treatment was used as a control. After 72 h, the luminescence was detected using a CTG kit. The EC50 and CC50 were calculated using Origin 9.1 software based on the luminescence readout.

### 4.4 In‐gel fluorescence analysis

RD cells were seeded in six‐well plates at 1 million cells per well for 12 h and then infected with EV‐A71 at an MOI of 0.01 PFU per cell for another 12 h. After treatment with indicated concentrations of MPA‐P and/or MPA‐CF_3_ for 0.5–4 h, the cells were washed with PBS and irradiated under UV light (UVP Crosslinker CL‐1000L) at 365 nm for 5 min on ice. The labeled cells were lysed with NP‐40 lysis buffer (Applygen) for 10 min on ice, and the supernatants were collected by centrifugation at 20,000×*g* at 4°C for 30 min followed by precipitation with chloroform–methanol. The recovered proteins were solubilized in 100 µL of 0.4% SDS/PBS by sonication. After determination of protein concentration with a BCA protein quantification kit (Applygen), the protein solution was adjusted to 1.8‐2 mg/mL with 0.4% SDS/PBS. Then click‐chemistry master mix was prepared by adding the reagents sequentially: 120 µL of 1.7 mM Tris((1‐benzyl‐1H‐1,2,3‐triazol‐4‐yl)methyl)amine (TBTA), 40 µL of 50 mM CuSO_4_, 40 µL of 50 mM Tris(2‐carboxyethyl)phosphine (TCEP) and 10 µL of 20 mM TAMRA‐N_3_. The click‐chemistry reaction was initiated by adding 10 µL of master mix to 100 µL of protein for 1 h at 29°C in the dark with gentle shaking (Eppendorf; ThermoMixer C). The reaction samples were mixed with 5× SDS loading buffer and separated by 12% SDS‒PAGE. The fluorescence of protein lanes and Coomassie brilliant blue (CBB) staining were visualized on the Bio‐Rad ChemiDoc MP imaging system.

### 4.5 Stable isotope dimethyl labeling

Three different isotopomers of formaldehyde were used for triplex stable isotope dimethyl labeling according to previous report.^52^ The preparation of digested samples for isotope labeling is list in Method S2, Supporting Information. For dimethyl labeling, CH_2_O (4 µL, 4%, “light” for labeling group), CD_2_O (4 µL, 4%, “medium” for competition group), and ^13^CD_2_O (4 µL, 4%, “heavy” for control group) were added to 100 µL of each digested sample. Then, 4 µL of 600 mM NaBH_3_CN (light and medium) and 4 µL of 600 mM NaBD_3_CN (heavy) were added. The solution was incubated at 25°C for 2 h with shaking. The reaction was quenched with 16 µL of 1% (v/v) ammonia followed by 8 µL of 5% formic acid (FA). After the solution was vortexed and separated through centrifugation, the three groups of labeled peptide solutions were pooled at a ratio of 1:1:1, subjected to desalination, dried in a vacuum centrifuge and reconstituted in 20 µL of buffer (95% water, 5% acetonitrile, 0.1% FA) for LC‒MS/MS analysis.

### 4.6 LC‒MS/MS and data analysis

LC‒MS/MS was performed on a Q‐Exactive Orbitrap mass spectrometer (Thermo Fisher Scientific) coupled with an Ultimate 3000 LC system. The mobile phase A contains 0.1% FA in water, and the mobile phase B contains 0.1% FA in acetonitrile. The loading rate was 3 µL/min and 0.3 µL/min for eluting. MS2 data collection was acquired using one full scan (350–1800 MW) followed by data‐dependent MS2 scans of the 20 most intense ions with dynamic exclusion enabled. The RAW Xtractor was used to extract the MS/MS spectra from the raw file, which were transformed into a ms2 format and were searched by the ProLuCID algorithm using the Human UniProt database (release‐2012_11). The customized software CIMAGEM was used to quantify the ratios of Light/Medium/Heavy.^52^

### 4.7 Pulldown/WB analysis of competition labeling

For each of the three experimental groups, RD cells were seeded in three 100‐mm dishes at 8 million cells per dish for 24 h and then infected with EV‐A71 at an MOI of 0.01 PFU per cell for another 12 h. The infected cells were treated with MPA‐P or cotreated with MPA‐CF_3_ for 1 h followed by photo‐crosslinking, cell lysis, protein precipitation, and resuspension in 0.4% SDS/PBS according to the steps of in‐gel fluorescence analysis. After the protein concentration was adjusted to 2 mg/mL, 1 mL of protein solution was reacted with 100 µL of click‐chemistry master mix (1.7 mM TBTA, 50 mM CuSO_4_, 50 mM TCEP and 20 mM biotin–PEG3–azide) at 29°C for 1 h. After the click reaction, the proteins were extracted by precipitation, solubilized in 1.2 mL of 1.2% SDS/PBS, and diluted in 4 mL of PBS. The dilutions were incubated with pretreated streptavidin magnetic beads (MCE) at 4°C for 4 h with gentle rotation. The beads were then washed with PBS and dissolved in 1× SDS loading buffer. After the beads were heated at 100°C for 8 min, the supernatants were collected with a magnetic stand and separated by 12% SDS‒PAGE for WB analysis.

### 4.8 WB analysis

The treated RD cells were lysed with NP‐40 lysis buffer containing protease inhibitor (Thermo Scientific; 78430) for 12 min on ice. The supernatants were extracted by centrifugation at 20,000×*g* at 4°C for 20 min. The protein concentrations were quantified and normalized. The protein samples were dissolved in 5× SDS loading buffer and denatured at 100°C for 8 min. The resulting samples or samples from the pulldown experiment were subjected to 12% SDS‒PAGE, and proteins were then transferred onto PVDF membranes (Millipore). The membranes were blocked with 5% skim milk (Beyotime) for 1 h at room temperature (RT) and then incubated with the corresponding primary antibody and horseradish peroxidase‐conjugated secondary antibody. Finally, the membranes were washed with TBS containing 0.1% Tween‐20 (TBST) and imaged on the Bio‐Rad ChemiDoc MP imaging system. The intensity of the images was analyzed with Image‐Pro Plus 6 software. The antibodies used are as follows: anti‐COPZ1 (1:2000; Proteintech; 20440‐1‐AP), anti‐EV‐A71 2C (1:2000, GTX132354), anti‐alpha Tubulin (1:5000; Abcam; ab7291), anti‐GAPDH (1:5000; Abcam; ab8245), anti‐beta actin (1:5000; Abcam; ab8226), anti‐EV‐A71 VP1 (1:1000; a gift from Professor Tong Cheng^53^), goat anti‐mouse IgG H&L (1:5000, ZB‐2305), and goat anti‐rabbit IgG H&L (1:5000; Abcam; ab6721).

### 4.9 Protein expression and purification

The synthetic gene of human COPZ1 encoding 7−150 amino acids with an N‐terminal 6×His‐TEV tag was cloned into a pET‐28A (+) expression vector using 5′ *Nco*I and 3′ *Xho*I sites. BL21(DE3) Chemically Competent Cells (TransGen) transformed with the pET‐28A (+)‐COPZ1 expression vector were propagated in LB medium at 37°C. The inducer isopropyl‐β‐d‐1‐thiogalactopyranoside (0.5 mM) was added to induce COPZ1^7‐150^ expression when an OD_600_ of cells reached 0.6–0.9. Then cells were incubated for 16 h at 18°C and separated by centrifugation at 4000×*g* at 4°C for 30 min. The pellets were suspended in lysis buffer (50 mM Tris‐HCl, pH 8.0, 300 mM NaCl, 10% glycerol, 1 mM TCEP, Halt Protease Inhibitor, 0.1% Triton X‐100) and lysed by sonication on ice for 6 min at 30% amplitude (SONICS). The supernatant was extracted by centrifugation at 20,000×*g* at 4°C for 30 min and applied on to a Ni‐NTA column (Sangon Biotech). The COPZ1 protein was eluted by elution buffer (50 mM Tris–HCl, 300 mM NaCl, 100 mM imidazole, pH 8.0). The COPZ1 fractions were further purified using a Sephadex G‐75 medium (Solarbio) in elution buffer (50 mM Tris–HCl, 300 mM NaCl, pH 8.0). The pure fractions were pooled and concentrated using Amicon Ultra Centrifugal Filters (Millipore; UFC8003). The protein purity was detected by CBB staining.

### 4.10 SPR analysis

COPZ1^7‐150^ was immobilized manually up to 8600 response units on the designated flow cell of sensor chip CM5 (Cytiva) on a Biacore S200 system. MPA‐CF_3_ was diluted in PBS‐P20 buffer (Cytiva) by a double dilution for 11 gradients. Solvent calibration was performed. The flow rate was set to 10 µL per min, and the injection time and dissociation time were set to 2 min. The *K*_d_ values were determined by the manufacture's software.

### 4.11 siRNA transfection

Reverse transfection of RNAi was performed to knockdown COPZ1 according to the manufacturer's protocol. Briefly, synthetic 100 nM COPZ1 siRNA and Lipofectamine RNAiMAX transfection reagent (Invitrogen; 13778‐150) were mixed in Opti‐MEM I (Gibco) to prepare RNA–lipid complexes and added to 24‐well plates. The suspended RD cells were mixed with the complexes in the wells. After incubation for 24 and 48 h, qRT‐PCR and WB were used to verify the transfection efficiency. After RD cells were transfected for 24 h, cells were infected with EV‐A71 at an MOI of 0.01 PFU per cell and treated with or without MPA‐CF_3_ (0.07 µM) or MPA‐P (0.07 µM) for 48 h. The viral RNA, supernatant, and proteins were extracted to evaluate the biological engagement of COPZ1. The well‐established COPZ1 siRNA target sequences^54^ and negative control sequences were synthesized by Tsingke Biotech (Beijing) and are listed in Table S2.

### 4.12 qRT‒PCR assay

The total RNA from the treated RD cells was extracted with TRI reagent (Sigma; T9424). Relative q RT‐PCR was used to determine the transfection efficiency with a One Step TB Green PrimeScript RT‐PCR Kit (TaKaRa; RR066A) on a QuantStudio 5 system (Thermo Fisher Scientific) according to the manufacturer's instructions. The relative mRNA expression was calculated using the 2^−△△Ct^ method with ACTB as an internal reference gene. The quantification of EV‐A71 RNA copies was performed by a FAM‐labeled probe with a One Step PrimeScript RT‐PCR Kit (TaKaRa; RR064A) on a QuantStudio 5 system. The viral RNA copies were calculated from the cycle threshold (Ct) values of samples on the corresponding standard curve and expressed as log(10) copies/µL. All primers of the detected genes and the FAM‐labeled probe,^55^ as well as plasmid standard sequence, are listed in Table S3.

### 4.13 Determination of TCID_50_

The supernatants from the siRNA transfection were collected and diluted serially 10 times in DMEM‐2% FBS, and the diluted sample (50 µL per well) were added to four wells of a 96‐well plate preseeded with Vero cells at 10^4^ cells per well. Cytopathic effect was recorded through 4−7 days of incubation at 37°C. The values of TCID_50_ per mL were calculated through a method by Reed and Muench.^56^

### 4.14 Immunofluorescence imaging

RD cells were transfected with siCOPZ1 and siNC and seeded on glass coverslips for 24 h. The transfected cells were infected with EV‐A71 at an MOI of 0.01 PFU per cell and treated with or without MPA‐CF_3_ (0.07 µM) for 48 h. Then cells were washed twice with PBS and fixed with 4% paraformaldehyde for 15 min. Subsequently, the cells were permeabilized with 0.5% Triton X‐100 in PBS for 20 min at RT and washed with PBS. Then, the cells were blocked with 3% bovine serum albumin (BSA) in TBST for 1 h and incubated with mouse anti‐EV‐A71 VP1 antibody (1:1000) at 4°C overnight. The cells were washed and incubated with Alexa Fluor 546 donkey anti‐mouse IgG (H+L) (1:500; Thermo Fisher Scientific; A10036) for 1 h in the dark at RT. Hoechst 33342 (5 µg/mL; Thermo Fisher Scientific; H21492) was finally used to counterstain the nuclei. Fluorescence images were captured using a Leica DMi8 inverted fluorescence microscope.

### 4.15 Protein TSA

Protein TSAs were performed using 4 µM COPZ1^7‐150^ protein in HEPES buffer (20 mM HEPES, 150 mM NaCl, pH 7.5) incubated with 10 µM MPA‐CF_3_ or DMSO in the presence of 20× Sypro Orange (Sigma, S5692). The final sample volume was 20 µL. The fluorescence emission intensities were recorded on a QuantStudio 5 system at a ramp rate of 0.05°C per second from 25°C through 95°C. Data were fitted by Protein Thermal Shift software v1.4 (Applied Biosystems) to calculate the *T*_m_ of COPZ1. GraphPad Prism 8.0 software was used to plot the *T*_m_ curves.

### 4.16 Co‐IP analysis

RD cells were seeded in six‐well plates at 1 million cells per well for 12 h and then infected with EV‐A71 at an MOI of 1 PFU per cell for 1.5 h. Uninfected RD cells were set as a negative control. At 18 hpi, the cells were washed and lysed with NP‐40 lysis buffer containing Halt Protease Inhibitor on ice. The supernatants were extracted by centrifugation at 20,000 g at 4°C for 20 min. The protein concentrations were adjusted to 2 mg/mL. The Protein A+G magnetic beads (Beyotime, P2108) were incubated with anti‐COPZ1 antibody for 1.5 h at RT with gentle rotation. The beads were washed, separated with a magnetic stand, and were incubated with aliquots of the lysate containing MPA‐CF_3_ at indicated concentrations at 4°C for 12−14 h with gentle rotation. The precipitated proteins on beads were eluted with 1× SDS loading buffer by heating at 100°C for 8 min and detected by WB.

### 4.17 Confocal microscopy analysis

RD cells were seeded in 15‐mm dishes (NEST, 801002) at 0.18 million cells per dish for 12 h and treated with MPA‐CF_3_ (0.67 µM) for 2 h followed by infection with EV‐A71 at an MOI of 1 PFU per cell for 1.5 h. At 8 hpi, cells were washed and fixed with 4% paraformaldehyde for 15 min and permeabilized with 0.3% Triton X‐100 in PBS for 20 min at RT. The cells were washed with PBS, blocked with 3% BSA in TBST for 1 h and incubated with anti‐EV‐A71 2C antibody (1:1000) and mouse anti‐Calnexin antibody (1:1000; Proteintech; 66903‐1‐Ig) for 1 h at RT. After washing with TBST, the cells were incubated with fluorescent secondary antibodies (1:500; Thermo Fisher Scientific; A10040 and A21202) for 1 h in the dark at RT. The nuclei were counterstained with Hoechst 33342. Fluorescence images were captured using a Leica SP8 STED confocal microscope.

### 4.18 Data analysis

All experimental data are presented as mean ± standard deviation (SD) and were analyzed with appropriate statistical tests specified in the methods and in the figure legends. Statistical differences were compared by GraphPad Prism 8.0 software. *p* < 0.05 was considered statistically significant.

## AUTHOR CONTRIBUTIONS

*Conceptualization of the study*: Yong Qin. *Research supervision*: Chu Wang and Wu Zhong. *Funding acquisition*: Song Li and Wu Zhong. *Probe synthesis, target identification, virology experiments, data collection and analysis, and writing the original draft*: Xiaoyong Li. *Quantitative chemoproteomics methodology*: Jin Zhang. *Preparation of MPA‐CF_3_*: Yaxin Xiao. *Review and editing of the manuscript*: Hao Song, Yuexiang Li, Wei Li, and Ruiyuan Cao. All authors read and approved the final manuscript.

## CONFLICT OF INTEREST STATEMENT

The authors declare that they have no conflict of interest.

## ETHICS STATEMENT

No ethical approval was necessary for this study.

## Supporting information

Supporting Information

Supporting Information

## Data Availability

The datasets used and/or analyzed in the study are available in the supplements or from the corresponding author upon reasonable request.
