# Supplementary material for: Chemoproteomics enables identification of coatomer subunit zeta‐1 targeted by a small molecule for enterovirus A71 inhibition
Source: MedComm (2020). 2024 Jun 5;5(6):e587. doi: 10.1002/mco2.587 (PMC11151152; doi:10.1002/mco2.587)
Supplement: Supplementary file 1 — Supporting Information [file MCO2-5-e587-s001.docx]

**Chemoproteomics enables identification of coatomer subunit zeta-1 targeted by a small molecule for enterovirus A71 inhibition**

Xiaoyong Li^1,2,4^, Jin Zhang^3,4^, Yaxin Xiao^1^, Hao Song^1^, Yuexiang Li^2^, Wei Li^2^, Ruiyuan Cao^2^, Song Li^2^, Yong Qin^1*^, Chu Wang^3*^ and Wu Zhong^2*^

^1^Key Laboratory of Drug-Targeting and Drug Delivery System of the Education Ministry, Sichuan Engineering Laboratory for Plant-Sourced Drug, and Sichuan Research Center for Drug Precision Industrial Technology, West China School of Pharmacy, Sichuan University, Chengdu 610041, China.

^2^National Engineering Research Center for the Emergence Drugs, Beijing Institute of Pharmacology and Toxicology, Beijing 100850.

^3^College of Chemistry and Molecular Engineering, Peking University, Beijing, China. ^4^These authors contributed equally: Xiaoyong Li, Jin Zhang.

^*^Corresponding authors: yongqin@scu.edu.cn; chuwang@pku.edu.cn; zhongwu@bmi.ac.cn

Supplementary Information

Method S1: Detailed procedure for the synthesis of compound **2**, **3**, **4** and **MPA-P**

Supplementary Note: Reactions required for anhydrous conditions were performed in ﬂame-dried glassware under argon atmosphere and all reagents were purchased from commercial suppliers. Reactions were monitored by thin layer chromatography (TLC). The synthetic products were purified by column chromatography on silica gel (200–300 meshes) or by preparative LC, and determined by NMR spectrometer. ^1^H NMR and ^13^C NMR spectra were recorded on Varian INOVA-400/54 and Agilent DD2-600/54 spectrometer, in CDC_l3_ solvent (reference peaks: ^1^H NMR: 7.26 ppm; ^13^C NMR: 77.16 ppm). High-resolution mass spectra (HRMS) were recorded on Bruker Apex IV FTMS or Agilent LC-MSD TOF ESI mass spectrometers.

Abbreviations

DCM: dichloromethane

TEA: triethylamine

MsCl: methanesulfonyl chloride

PE: petroleum ether

EA: ethyl acetate

MeOH: methanol

NaHCO_3_: sodium bicarbonate

K_2_CO_3_: potassium carbonate

DMF: *N,N*-dimethylformamide

TLC: thin layer chromatography

RT: room temperature

min: minutes

h: hours

The synthesis of compound **2**

Compound **1** (2.30 g, 16.7 mmol) was dissolved in dry DCM (300 mL). The mixture was stirred at 0 °C for 10 min, and TEA (9.26 mL) was added, followed by dropwise addition of MsCl (2.58 mL). The mixture was stirred at RT for 8 h. TLC showed that the reaction was completed. The reaction was quenched with water. The mixture was extracted with DCM (3 × 100 mL). The combined organic layers were dried with anhydrous sodium sulfate and concentrated under vacuum. The crude material was purified by column chromatography (PE: EA = 20:1) to afford compound **2**.

The synthesis of compound **3**

Compound **2** (2.76 g, 128 mmol) and potassium thioacetate (3.21 g, 281 mmol) were dissolved in acetone (500 mL) under argon. The mixture was stirred at RT for 12 h. TLC showed that the reaction was completed. Water was added to dissolve the reaction mixture. The mixture was extracted with DCM (2 × 100 mL). The combined organic layers were dried with anhydrous sodium sulfate and concentrated under vacuum. The crude material was purified by column chromatography (PE: DCM = 40:1) to afford compound **3**.

The synthesis of compound **4**

Compound **3** (2.35 g, 12.0 mmol) was dissolved in dry MeOH (100 mL), and hydrazine hydrate solution (80%, 1 mL) was added dropwise at RT. The mixture was stirred at RT for 4 h. TLC showed that the reaction was completed. The reaction was quenched with saturated aqueous NaHCO_3_ and extracted with EA (3 × 100 mL). The combined organic layers were dried with anhydrous sodium sulfate and concentrated under vacuum. The crude material was purified by column chromatography (PE: EA = 20:1) to afford side chain **4**.

The synthesis of **MPA-P**

Compound **4** (1.03 g, 6.7 mmol), **MPA-CF_3_** (0.43 g, 0.67 mmol) and anhydrous K_2_CO_3_ (0.93 g, 6.7 mmol) were dissolved in extra dry DMF (100 mL) under argon. The mixture was stirred at 80 °C for 72 h. The reaction was quenched with water and extracted with EA (3 × 100 mL). The combined organic layers were dried with anhydrous sodium sulfate and concentrated under vacuum. The crude material was purified by column chromatography (PE: EA = 4:1) and by preparative LC to afford the photoaffinity probe **MPA-P**.

**Characterization data: ^1^H NMR Spectra, ^13^C NMR Spectra of all compounds**

Compound **2**: ^1^H-NMR (600 MHz, CDCl_3_) δ 4.08 (t, *J* = 6.2 Hz, 2H), 3.06 (s, 3H), 2.06 – 1.99 (m, 3H), 1.90 (t, *J* = 6.2 Hz, 2H), 1.70 (t, *J* = 7.8 Hz, 2H); ^13^C-NMR (150 MHz, CDCl_3_) δ 82.5, 69.6, 64.1, 37.7, 33.0, 32.3, 26.0, 13.3.

Compound **3**: ^1^H-NMR (600 MHz, CDCl_3_) δ 2.69 (t, *J* = 7.8 Hz, 2H), 2.32 (s, 3H), 2.06 – 1.97 (m, 3H), 1.73 – 1.64 (m, 4H); ^13^C-NMR (150 MHz, CDCl_3_) δ 195.3, 82.6, 69.4, 33.3, 32.1, 30.7, 27.7, 23.5, 13.4.

Compound **4**: ^1^H-NMR (400 MHz, CDCl_3_) δ 2.49 – 2.40 (m, 2H), 2.06 – 1.99 (m, 3H), 1.86 – 1.78 (m, 2H), 1.66 (t, *J* = 7.2 Hz, 2H); ^13^C-NMR (100 MHz, CDCl_3_) δ 82.6, 69.5, 32.7, 32.4, 32.3, 27.6, 13.4.

**MPA-P**: ^1^H-NMR (400 MHz, CDCl_3_) δ 8.01 (d, *J* = 8.4 Hz, 1H), 7.57 (d, *J* = 8.4 Hz, 1H), 7.36 – 7.32 (m, 1H), 7.25 – 7.17 (m, 2H), 6.85 (s, 1H), 6.66 – 6.60 (m, 1H), 2.37 – 2.28 (m, 1H), 2.20 – 2.10 (m, 1H), 2.00 – 1.96 (m, 1H), 1.94 – 1.88 (m, 2H), 1.53 – 1.32 (m, 4H); ^13^C-NMR (100 MHz, CDCl_3_) δ 187.1, 185.0, 162.8, 160.6, 137.6 (q, *J* = 33 Hz), 136.8 (q, *J* = 33 Hz), 132.9, 130.9, 130.9, 130.5, 124.6, 124.1, 124.1, 123.1 (q, *J* = 272 Hz), 123.0 (q, *J* = 272 Hz), 122.7, 121.3, 121.1, 120.9, 116.1, 115.7, 115.2, 115.0, 110.2, 82.5, 69.5, 33.0, 32.0, 32.0, 27.2, 13.3. HRMS (ESI) calculated for C_31_H_18_Cl_3_F_6_N_4_O_4_S [M-H]^-^: 761.0024, found 761.0024.

**Characterization data: HRMS of MPA-P**


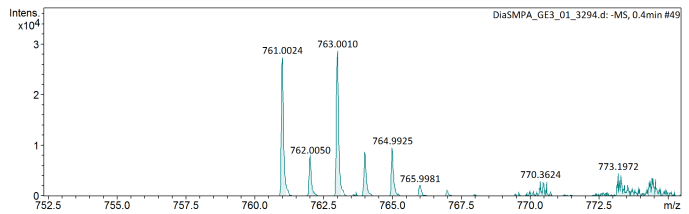


Method S2: The preparation of digested samples for stable isotope dimethyl labeling

As illustrated in Figure 3A, three experimental groups were performed in parallel for proteome analysis. For each group, RD cells were seeded in three 100-mm dishes (Corning) at 10 million cells per dish for 24 h and then infected with EV-A71 at an MOI of 0.01 PFU per cell for another 12 h. The infected cells were treated with MPA-P or cotreated MPA-CF_3_ for 1 h followed by photo-crosslinking, cell lysis, protein precipitation and resuspension in 0.4% SDS/PBS according to the steps of in-gel fluorescence analysis. The resulting proteins were diluted to 2 mg/mL and delivered 1 ml to react with 100 µL of a click-chemistry master mix (1.7 mM TBTA, 50 mM CuSO_4_, 50 mM TCEP and 20 mM biotin-PEG3-azide) for 1 h at 29 °C. After the click reaction, the proteins were precipitated, resuspended in 1 mL of 1.2% SDS/PBS and diluted 5-fold with PBS. The dilutions were incubated with pretreated streptavidin beads (Thermo Fisher Scientific) at 29 °C for 4 h with gentle rotation. The beads were then washed sequentially with PBS and water before being transferred to the Protein LoBind tubes (Eppendorf). The enriched proteins were subjected to denaturation, disulfide bond reduction and alkylation. Then, the beads were washed and collected by centrifugation. The proteins on beads were digested with a premix of 200 µL of 1 M urea in 100 mM triethyl ammonium bicarbonate (TEAB)/PBS buffer, 2 µL of 100 mM calcium chloride in water and 4 µL of trypsin (0.5 µg/mL, Promega) at 37 °C for 12 h with gentle rotation. Finally, 200 µL of each digested sample was transferred to Protein LoBind tubes by centrifugation for stable isotope dimethyl labeling.

Table S1: The selected nine proteins for target identification

| UniProt Entry | Protein names | Mass (Da) |
| --- | --- | --- |
| P13073 | Cytochrome c oxidase subunit 4 isoform 1, mitochondrial | 19,577 |
| P61923 | Coatomer subunit zeta-1 | 20,198 |
| O60831 | PRA1 family protein 2 | 19,258 |
| P67812 | Signal peptidase complex catalytic subunit SEC11A | 20,625 |
| Q9Y3E5 | Peptidyl-tRNA hydrolase 2, mitochondrial | 19,194 |
| P61009 | Signal peptidase complex subunit 3 | 20,313 |
| P40616 | ADP-ribosylation factor-like protein 1 | 20,418 |
| Q15382 | GTP-binding protein Rheb | 20,497 |
| Q9Y584 | Mitochondrial import inner membrane translocase subunit Tim22 | 20,031 |

Table S2: COPZ1 siRNA target sequences (siCOPZ1) and negative control sequences (siNC)

| Gene names | siRNA sequences |
| --- | --- |
| human siCOPZ1 | Sense: GGACAAUGAUGGAGAUCGATT |
| human siCOPZ1 | Anti-sense: UCGAUCUCCAUCAUUGUCCTT |
| Negative control (siNC) | Sense: UUCUCCGAACGUGUCACGUTT |
| Negative control (siNC) | Anti-sense: ACGUGACACGUUCGGAGAATT |

Table S3: Primers, labeled probe and plasmid standard sequence

| Gene names | Commercially synthesized sequences |
| --- | --- |
| human ACTB | forward: 5′-GGCATCCTCACCCTGAAGTA-3′ |
| human ACTB | reverse: 5′-AGAGGCGTACAGGGATAGCA-3′ |
| human COPZ1 | forward: 5′-CAGTGAAATTGCCCTCTTGGA-3′ |
| human COPZ1 | reverse: 5′-TCAGAACAGCCATAAGCATCAG-3′ |
| EV-A71 | forward: 5′-CCAATCTCAGCGGCTTGGAG-3′ |
| EV-A71 | reverse: 5′-CACTCAAGCTCTACCGGCAC-3′ |
| FAM-labeled probe | 5′-(6-FAM)-TCCAATCGATGGCTGCTCACCTGCGT-(BHQ1)-3′ |
| Plasmid standard sequence | CCAATCTCAGCGGCTTGGAGTGCTGGGACTTTACCAGTGTCCAATCGATGGCTGCTCACCTGCGTGTTCTGACCTGTGGGTGCCGGTAGAGCTTGAGTG |

Figure S1: The cytotoxic effects of MPA-CF_3_, HCQ and MG-132 on RD cells

**
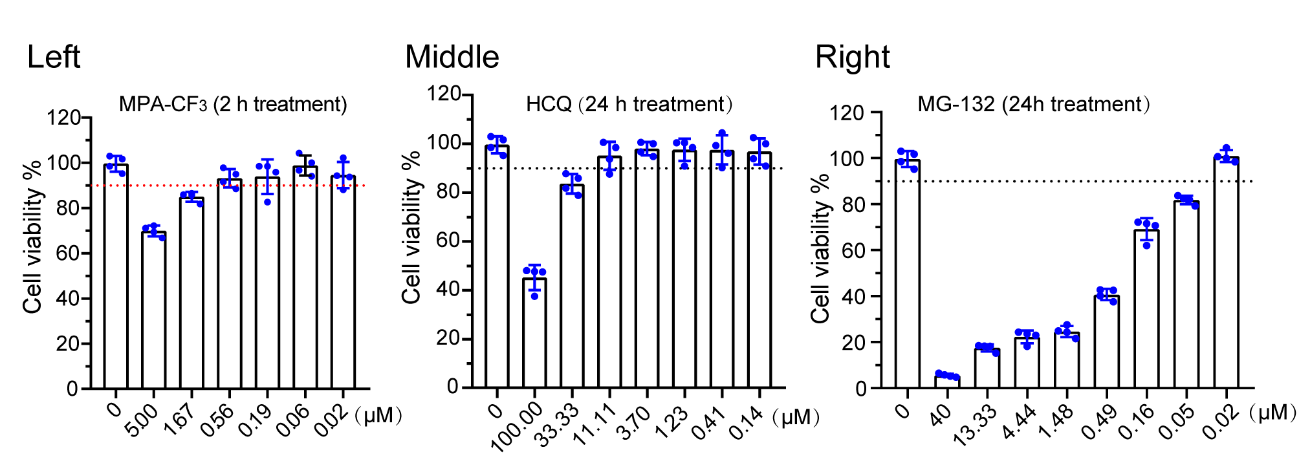
**

RD Cells were treated with the indicated concentrations of MPA-CF_3_ for 2h, hydroxychloroquine (HCQ) and MG-132 for 24h, respectively. Then the cell viabilities were measured by using a CTG kit. The dashed line represents 90% cell viability. Data are presented as the mean ± SD of four biological replicates.
